# Circulating levels of cell adhesion molecules and risk of cardiovascular events in obstructive sleep apnea

**DOI:** 10.1371/journal.pone.0255306

**Published:** 2021-07-30

**Authors:** Bernardo U. Peres, A. J. Hirsch Allen, Patrick Daniele, Karin H. Humphries, Carolyn Taylor, Ismail Laher, Fernanda Almeida, Rachel Jen, Andrew J. Sandford, Stephan F. van Eeden, Najib T. Ayas

**Affiliations:** 1 Department of Oral Health Sciences, Faculty of Dentistry, University of British Columbia, Vancouver, Canada; 2 Department of Medicine, Faculty of Medicine, University of British Columbia, Vancouver, Canada; 3 Centre for Improved Cardiovascular Health, (CHÉOS), Vancouver, Canada; 4 Division of Cardiology, Department of Medicine, Faculty of Medicine, University of British Columbia, Vancouver, Canada; 5 Canadian Sleep and Circadian Network, Montréal, Canada; 6 Centre for Heart Lung Innovation, St. Paul’s Hospital, University of British Columbia, Vancouver, Canada; 7 Leon Judah Blackmore Sleep Disorders Program, UBC Hospital, Vancouver, Canada; Harper University Hospital, UNITED STATES

## Abstract

**Background:**

Obstructive sleep apnea (OSA) patients are at increased risk of cardiovascular disease (CVD). Cell adhesion molecules (CAM) are increased in OSA and CAM are also implicated in the development of CVD.

**Research question:**

Do CAM (ICAM-1, VCAM-1 and E-selectin) have prognostic value in identifying risk of cardiovascular events in OSA?

**Study design and methods:**

Patients with suspected OSA referred for a polysomnogram provided a fasting blood sample. Plasma levels of ICAM-1, VCAM-1 and E-selectin were determined by multiplex Luminex Assay (Milliporesigma ON, Canada). Cardiovascular events were determined by deterministic linkage to provincial health databases.

**Results:**

418 patients were included in the analysis. Mostly male (68.2%), mean age of 50.7 yrs, median AHI 16.5 events/hour, and mean BMI of 31.7 kg/m^2^. 36 cardiovascular events occurred in 8-yrs of follow up. Higher levels of ICAM-1 were associated with developing CVD (HR = 3.65 95% CI 1.40–9.53, 2^nd^ and 3rd tertiles vs. 1^st^ tertile), including in patients with OSA (HR = 3.1 95% CI 1.16–8.25). E-selectin was significantly associated with cardiovascular events in patients with moderate to severe OSA (HR = 3.31 95% CI 0.94–11.72, 2^nd^ and 3^rd^ tertiles vs. 1^st^ tertile) but not in patients without moderate to severe OSA (HR = 0.67 95% CI 0.19–2.38), p-value for interaction = 0.07.

**Interpretation:**

In a suspected OSA cohort, patients with higher levels of ICAM-1 (>816 ng/ml) were significantly more likely to experience a cardiovascular event within 8 years after PSG. In moderate to severe OSA patients, a higher E-selectin (>36.4 ng/ml) was significantly associated with cardiovascular events.

## Introduction

Obstructive Sleep Apnea (OSA) is the most common respiratory sleep disorder with close to half a billion people having moderate to severe disease globally [1]. Untreated OSA is associated with a significantly increased risk of cardiovascular disease (CVD) including stroke and myocardial infarction [2]; however, identifying patients with a particularly high risk has been challenging as standard metrics of OSA severity (such as the apnea hypopnea index) are not particularly discriminative. The ability to identify such a high-risk group could help direct more aggressive treatment of OSA and other CV risk factors (personalized or precision care) or facilitate patient selection for recruitment into clinical trials of CV prevention. Recent efforts have been made to utilize novel parameters to help identify a group at high risk of CVD [3]; these have included using symptom clusters [4], PSG clusters [5], comorbidities [6], and desaturation parameters [7] to help identify such patients.

Circulating levels of inflammatory markers may be a potentially useful method to risk stratify OSA patients [8]. Inflammation plays a pivotal role in the initiation and progression of CVD [9]. In addition, OSA is characterized by intermittent hypoxia and oxidative stress, which in turn activates systemic inflammation [10]; both animals exposed to intermittent hypoxia and patients with OSA have increased levels of inflammatory markers including C-reactive protein (CRP), interleukins, and cellular adhesion molecules [11–13]. This inflammation is likely a contributing factor to premature CVD in OSA patients.

Cell adhesion molecules (CAMs) are biomarkers of inflammation that might be particularly helpful in this regard. CAMs, such as endothelial selectin (E-selectin), vascular cell adhesion molecule-1 (VCAM-1), and intercellular adhesion molecule-1 (ICAM-1) increase binding and recruitment of white blood cells to the endothelium [14], contribute to atherosclerotic plaque development, and are linked to the occurrence of CVD [15–17]. Given that OSA can also increase CAM, we hypothesized that circulating CAM levels would be associated with future risk of CV events in OSA. The aim is that these molecules could contribute to establish a ‘biochemical phenotype’, and eventually help to risk stratify OSA patients in terms of CVD risk.

## Materials and methods

### Study design, setting and participants

This cohort has been used and previously described [18]. Briefly, consenting adults (>19 years old) referred for suspected OSA to the University of British Columbia Hospital Sleep Disorder Laboratory for inpatient polysomnography (PSG) were recruited from 2003 to 2008. Patients that were unable to speak English and being treated for OSA were excluded. On the night of their PSG, patients completed a detailed questionnaire about their medical history, sleep symptoms and habits.

Apneas and hypopneas were scored according to the recommendations of the American Academy of Sleep Medicine (AASM), where hypopneas were defined according to a reduction in airflow with either a 3% desaturation or an arousal from sleep [19]. Patients were diagnosed as having OSA based on an AHI of ≥5 events/hour. An AHI between 5 and 15 events/hour was considered mild, ≥15–30 events/hour was considered moderate and ≥30 events/hour was considered severe OSA [19].

This study was approved by the University of British Columbia Research Ethics Board (H13-00346) and Vancouver Coastal Health Research Institutes (V11-80199).

### Laboratory analysis

Fasting blood (15 ml) was collected on the morning after PSG, centrifuged, and stored in a -80°C freezer. Endothelial selectin (E-selectin), vascular cell adhesion molecule-1 (VCAM-1), and intercellular adhesion molecule-1 (ICAM-1) were measured using Milliplex Map Human Cardiovascular Disease Panel 1 multiplex Luminex assay (Milliporesigma ON, Canada) from frozen plasma samples. Manufacturer’s protocol was followed, and all measurements done in duplicates.

### Ascertainment of cardiovascular events

The major outcome of interest was a composite of incident cardiovascular and cerebrovascular events which included cardiovascular death, hospitalization for cardiovascular conditions, stroke and cardiac procedures (percutaneous coronary intervention-PCI, coronary artery bypass graft- CABG); follow-up time was 8 years from PSG date for all patients. The outcomes were obtained by linking our cohort to provincial health databases as has been done in previous studies [8]. The codes indicating deaths from cardiovascular-related causes are summarized in S1 Table in S1 File; hospitalizations, procedures and events codes and definitions are in S2 Table in S1 File. These events were identified by deterministic linkage of consenting patients to different provincial health databases through Population Data BC (PopdataBC). For more details on the provincial databases used and PopdataBC disclaimers, please refer to the supplemental section of the paper. The coding for the databases used have been previously validated [20].

Comorbidities and potential confounding were determined based on self-reports from health questionnaires. History of previous cardiovascular disease (CVD) was determined based on previous doctors diagnoses of hypertension, myocardial infarction, cardiac arrhythmias, angina, and congestive heart failure. Smoking status (currently smoking versus not currently smoking), usage of statins, and presence of diabetes were also self-reported.

Only BC residents were included in the cohort. To be considered a BC resident, we required continuous provincial health registration with no larger than a 93-day (~ 3 month) gap in registration following PSG date.

### Continuous positive airway pressure adherence

CPAP adherence was determined by chart review. Two independent trained researchers (BP and AHA) reviewed all charts for objective and subjective data on CPAP adherence. CPAP providers’ reports and patient reports dictated in the physicians’ notes were used to determine adherence. Adherence was defined as minimum of 4 hours per night for at least 70% of the nights [21]. In the absence of objective measures, physician notes indicating a clear positive response to CPAP prescription and usage were considered as adherent to treatment. Non-adherence was defined as: reported use below 4 hours per night for at least 70% of the nights, clear intolerance to CPAP, and failed to return for a follow-up consultation after being prescribed CPAP. Data regarding patient’s adherence was charted up to 60 days from the CPAP prescription day. CPAP was usually prescribed within a week of the PSG. The data was plotted and scored independently. Interrater reliability was excellent (kappa value of 0.99).

### Statistical analysis

Continuous variables that were normally distributed were summarized with mean and standard deviations then tested with t-tests. Continuous variables that were skewed were summarized with medians and IQRs then tested with Mann-Whitney-U tests. Categorical variables were summarized with counts and proportions and compared with Chi-square tests when all expected cell counts were >5 or Fisher’s exact tests otherwise.

Associations of baseline variables and occurrence of cardiovascular events were modelled using Cox proportional hazards models to estimate hazard ratios with 95% confidence intervals; only first events were used in the analysis. We assessed univariate associations of adhesion molecules and comorbidities with cardiovascular events then constructed final adjusted models. Levels of adhesion molecules were divided in tertiles (as opposed to quartiles) to minimize excessive data segmentation. Also, preliminary analysis dividing the data in quartiles yielded similar results (S3 Table in S1 File).

Adjusted models included age, sex, AHI, body mass index (BMI), previous heart disease, diabetes, Epworth Sleepiness Scale (ESS), statin usage, CPAP adherence and smoking status (current smokers vs. non-current smokers) as these variables were felt to be important confounders. Due to the high correlation between oxygen desaturation and AHI and the fact that replacement of AHI with desaturation did not change results appreciably, AHI was the variable used in the models as a measure of OSA severity. For CPAP usage adjustment, a three-level categorization was created; patients were classified as prescribed and adherent, prescribed and non-adherent, and not prescribed.

As an exploratory analysis, we determined whether the association between adhesion molecule levels and cardiovascular events differed according to sleep apnea severity. That is, we performed an interaction analysis, using fully adjusted models, based on AHI thresholds of 5 and 15 (i.e. any OSA, and moderate to severe OSA) to determine if hazard ratios differed. Previous literature supported the use of a level of significance of 20% (p<0.2) in exploratory interaction analysis [22]. To be more stringent we set the significance level at 10% (p<0.1) for this exploratory analysis.

We used similar descriptive and inferential statistics to investigate the association between CPAP adherence and cardiovascular events in this cohort. For this analysis, only patients prescribed CPAP were included.

Cell sizes <6 were censored to protect patient privacy and comply with Population Data BC regulations regarding small sizes. Statistical Analysis Software (SAS version 9.4, SAS Institute Inc, USA) was used.

## Results

A total of 1983 patients were recruited in the cohort, of those 488 patients had CAM levels measured. Of these, 70 did not have adequate follow up due to BC non residence or lack of consent for data linkage. The final number of patients recruited for this study was, thus, 418 (Table 1). Most of the patients were Caucasian (80%) and the majority were male (68.7%), with a mean age of 50.7 years. Median AHI was 16.5/hr and mean BMI was 31.7 kg/m^2^.

**Table 1:** Patient characteristics by adhesion molecule tertiles (n = 418).

	*Overall Cohort*	*ICAM-1*				*VCAM-1*				*E-selectin*			
*Baseline characteristics*	*(n = 418)*	*Tertile 1*	*Tertile 2*	*Tertile 3*	*P-value*	*Tertile 1*	*Tertile 2*	*Tertile 3*	*P-value*	*Tertile 1*	*Tertile 2*	*Tertile 3*	*P-value*
		*(n = 139)*	*(n = 140)*	*(n = 139)*		*(n = 139)*	*(n = 141)*	*(n = 138)*		*(n = 139)*	*(n = 140)*	*(n = 139)*	
Age (years)–mean ± SD	50.7 ± 11.5	52.0 ± 11.3	49.3 ± 11.6	50.9 ± 11.5	0.16	50.0 ± 11.2	52.4 ± 11.7	51.7 ± 12.1	0.30	51.0 ± 11.7	50.9 ± 11.6	50.3 ± 11.3	0.85
Sex (Male)–n (%)	287 (68.7)	91 (65.9)	97 (68.8)	99 (71.2)	0.64	92 (78.6)	88 (75.2)	61 (52.1)	**<0.001**	83 (60.1)	96 (68.6)	108 (77.1)	**0.009**
BMI (kg/m^2^)—mean ± SD	31.7 ± 6.5	30.3 ± 6.2	30.8 ± 5.5	33.9 ± 7.1	**<0.001**	31.2 ± 5.5	31.1 ± 5.7	32.4 ± 7.6	0.21	29.3 ± 5.8	32.4 ± 6.3	33.3 ± 6.6	**<0.001**
AHI—events/hr–median (IQR)	16.5 (7,30.4)	16 (7.4,30.0)	16.6 (6.9,28.1)	17.8 (7.1,35.4)	0.49	16.6 (8.2,34.8)	18.4 (7.9,30.9)	13.8 (5.4,26.8)	0.08	12.8 (6,24.3)	16 (7.1,30.1)	20.4 (8.2,38.9)	**<0.001**
AHI Category					0.42				**0.009**				**0.04**
No OSA–n (%)	68 (16.3)	17 (12.3)	27 (19.1)	24 (17.3)	.	15 (12.8)	14 (11.9)	29 (24.8)	.	26 (18.8)	22 (15.7)	20 (14.3)	.
Mild OSA—n (%)	127 (30.4)	49 (35.5)	39 (27.7)	39 (28.1)	.	40 (34.2)	39 (33.1)	31 (26.5)	.	51 (37)	41 (29.3)	35 (25.0)	.
Moderate OSA—n (%)	114 (27.3)	36 (26.1)	43 (30.5)	35 (25.2)	.	24 (20.5)	34 (28.8)	37 (31.6)	.	37 (26.8)	41 (29.3)	36 (25.7)	.
Severe OSA—n (%)	109 (26.1)	36 (26.1)	32 (22.7)	41 (29.5)	.	38 (32.5)	31 (26.3)	20 (17.1)	.	24 (17.4)	36 (25.7)	49 (35.0)	.
% Time below 90% SaO_2—_median (IQR)	0.32(0.1,2.5)	0.31(0.1,1.7)	0.2 (0,2.3)	0.7 (0.1,3.6)	**0.05**	0.5 (0.1,2.8)	0.3 (0.1,2.2)	0.3 (0.1,2.5)	0.49	0.2 (0,1.5)	0.4 (0.1,2.4)	0.6 (0.1,3.2)	0.30
ESS > 11 –n (%)	136 (30.1)	31 (22.3)	44 (31.2)	51 (36.7)	**0.03**	30 (25.6)	45 (38.1)	28 (23.9)	**0.03**	32 (23)	44 (31.4)	50 (35.7)	0.06
Heart Disease*—n (%)	90 (21.5)	26 (18.7)	29 (20.6)	35 (25.2)	0.40	32 (27.4)	31 (26.3)	22 (18.8)	0.25	28 (20.1)	29 (20.7)	33 (23.6)	0.76
Smoking Status^+^ - n (%)	37 (8.8)	9 (6.5)	8 (5.7)	20 (14.4)	**0.02**	9 (7.7)	10 (8.5)	6 (5.1)	0.58	9 (6.5)	11 (7.9)	17 (12.1)	0.22
Diabetes—n (%)	32 (7.6)	9 (6.5)	9 (6.4)	14 (10.1)	0.42	13 (11.1)	8 (6.8)	10 (8.5)	0.50	12 (8.6)	< 6	15 (10.7)	0.07
Ethnicity Group					0.06				0.81				0.06
Caucasian—n (%)	328 (80)	110 (82.1)	113 (80.7)	105 (77.2)	.	88 (77.9)	93 (81.6)	96 (84.2)	.	110 (80.9)	118 (86.8)	100 (72.5)	.
Asian—n (%)	36 (8.8)	16 (11.9)	11 (7.9)	9 (6.6)	.	13 (11.5)	10 (8.8)	9 (7.9)	.	12 (8.8)	8 (5.9)	16 (11.6)	.
Other—n (%)	46 (11.2)	8 (6)	16 (11.4)	22 (16.2)	.	12 (10.6)	11 (9.6)	9 (7.9)	.	14 (10.3)	10 (7.4)	22 (15.9)	.
Statin User—n (%)	80 (19.4)	26 (19.3)	25 (17.7)	29 (21.3)	0.75	25 (21.7)	20 (17.5)	25 (22.1)	0.64	23 (16.9)	32 (23.4)	25 (18)	0.35
Adhesion Molecules (ng/ml)	.	249.8–816.1	816.13–996.1	996.1–1908.0	.	8–60.1	60.1–83.4	83.41–352.7	.	9.7–36.4	36.41–52.1	52.11–220.8	.
Rate of 1^st^ Cardiovascular Event	36 (8)	<6 (3.6)	16 (11.4)	15 (10.7)		10 (7.19)	9 (6.3)	17 (12.3)		8 (5.7)	16 (11.4)	12 (8.6)	

***Abbreviations***: AHI: Apnea-Hypopnea Index; BMI: Body mass index; ESS: Epworth Sleepiness Scale; ICAM-1: Intercellular adhesion molecule-1; IQR: Interquartile range; SD: Standard deviation; SaO_2_: Oxygen Saturation; VCAM-1: Vascular Cell Adhesion Molecule-1; *Heart disease included: Hypertension, Myocardial Infarction; Cardiac Arrhythmias, Angina, and Congestive Heart Failure. ^+^Current Smokers.

There was a total of 36 first events included in the analysis (Table 2). The majority of first events were CV death, myocardial infarction, unstable angina, or percutaneous coronary intervention. Age, male sex, AHI, statin use, and diabetes were associated with higher rates of cardiovascular events (S4 Table in S1 File).

**Table 2:** Percentage of cardiovascular events (n = 36).

Outcome	First Event (%)
Cardiovascular Death*	13.9 (CVD)
Atrial Fibrillation	5.6
Congestive Heart Failure	8.3
Myocardial Infarction	19.4
Stroke	5.6
Unstable Angina	13.9
Coronary Artery Bypass Grafting	2.8
Percutaneous Coronary Intervention	19.4
Cardioversion	5.6
Pacemaker	5.6

*Causes of Death: Hyperlipidemia, myocardial infarction, atherosclerotic heart disease, chronic ischemic heart diseases and myocarditis (S1 Table in S1 File)

### Association of cardiovascular events with CAM levels

The baseline characteristics according to CAM tertiles are shown in Table 1. For ICAM-1 and E-selectin, BMI significantly increased across the tertiles. Greater AHI was associated with E-selectin tertiles (p<0.001). Increased subjective sleepiness was associated with higher ICAM-1 tertiles.

For ICAM-1 and E-selectin, rates of first cardiovascular events were similar in the 2^nd^ and 3^rd^ tertiles and greater than in the first. That is, the top two tertiles (2^nd^ and 3^rd^) of ICAM-1 had event rates of 11.4% and 10.7%, while the first tertile had an event rate of 3.6%. E-selectin 2^nd^ and 3^rd^ tertiles had event rates of 11.4% and 8.6%, respectively, while the 1^st^ tertile of E-selectin had an event rate of 5.7%. For VCAM-1, the rate in the 3^rd^ tertile (12.3%) was greater than the 1^st^ (7.2%) and 2^nd^ tertiles (6.3%).

The associations between CAM levels and cardiovascular events are shown in Table 3. For ICAM-1, we compared the highest two tertiles with the 1^st^ for the multivariable models. In fully adjusted models, a higher ICAM-1 level was independently associated with risk of cardiovascular events (HR 3.65, 95% CI 1.40–9.53, p = 0.00). Although hazard ratios were above one, VCAM-1 and E-selectin levels were not significantly associated with the incidence of cardiovascular events.

**Table 3:** Unadjusted and adjusted hazard ratios for adhesion molecule tertiles and cardiovascular events.

***ICAM-1***		
**Unadjusted Tertiles**	**HR (95% CI)**	**p-value**
**2 vs 1**	3.27 (1.20, 8.91)	**0.02**
**3 vs 1**	3.25 (1.18, 8.93)	**0.02**
**Tertile 2+3 vs 1**	**HR (95% CI)**	**p-value**
**Unadjusted**	3.26(1.27, 8.37)	**0.01**
Fully Adjusted***	3.65 (1.40, 9.53)	**0.008**
***VCAM-1***		
**Unadjusted Tertiles**	**HR (95% CI)**	**p-value**
**2 vs 1**	0.90 (0.37, 2.22)	0.83
**3 vs 1**	1.84 (0.84, 4.02)	0.13
**Tertile 2+3 vs 1**	**HR (95% CI)**	**p-value**
**Unadjusted**	1.30 (0.63, 2.72)	0.48
Fully Adjusted***	1.19 (0.53, 2.66)	0.67
***E-selectin***		
**Unadjusted Tertiles**	**HR (95% CI)**	**p-value**
**2 vs 1**	2.09 (0.90, 4.90)	0.09
**3 vs 1**	1.54 (0.63, 3.76)	0.34
**Tertile 2+3 vs 1**	**HR (95% CI)**	**p-value**
**Unadjusted**	1.81 (0.83, 3.98)	0.14
Fully Adjusted***	1.83 (0.80, 4.20)	0.15

***Adjusted for Age, Sex, Smoking Status, AHI, BMI, Heart Disease, ESS > 11, Diabetes, CPAP usage, Statin Usage

### Interaction with OSA severity

We compared hazard ratios of events according to CAM tertiles by varying degrees of OSA severity using an interaction analysis at a significance level of 10% (Table 4). We found that in OSA patients, first cardiovascular event rates were greater in the two higher tertiles of ICAM vs. the 1^st^ tertile (11.8% vs. 3.3%) with an adjusted HR similar to the entire cohort of suspected OSA (adjusted HR = 3.1, CI: 1.16–8.25). In patients without OSA and in the lowest tertile of ICAM-1, there were no cardiovascular events (0/17 patients) suggesting that the absence of OSA together with low ICAM levels is indicative of a very low risk group; non-OSA patients in the second and third tertiles of ICAM-1 experienced an event rate of 7.8%. When patients were stratified by the presence or absence of moderate to severe OSA, ICAM-1 was similarly predictive in both groups with no significant interaction (p = 0.50). There was no interaction with VCAM-1 levels and OSA severity.

**Table 4:** Stratified hazard ratios for cardiovascular events by AHI levels.

**With OSA versus Without OSA (AHI <5 versus AHI ≥ 5)**		
**ICAM-1 (2+3 vs 1)**	**HR (95% CI)**	**p-value for interaction**
2+3 vs 1 at AHI <5	Cannot be estimated	0.99
2+3 vs 1 at AHI ≥5	3.1 (1.16, 8.25)	
**VCAM-1 (2+3 vs 1)**	**HR (95% CI)**	**p-value for interaction**
2+3 vs 1 at AHI <5	1.95 (0.44, 8.67)	0.45
2+3 vs 1 at AHI ≥5	1.14 (0.51, 2.58)	
**E-selectin (2+3 vs 1)**	**HR (95% CI)**	**p-value for interaction**
2+3 vs 1 at AHI <5	0.34 (0.04, 2.62)	0.10
2+3 vs 1 at AHI ≥5	2.21 (0.87, 5.66)	
**With Moderate to Severe OSA versus Without Moderate to Severe OSA AHI <15 versus AHI ≥ 15**		
**ICAM-1 (2+3 vs 1)**	**HR (95% CI)**	**p-value for interaction**
2+3 vs 1 at AHI <15	6.10 (0.76, 48.64)	0.50
2+3 vs 1 at AHI ≥15	2.74 (0.91, 8.20)	
**VCAM-1 (3 vs 1+2)**	**HR (95% CI)**	**p-value for interaction**
3 vs 1+2 at AHI <15	0.81 (0.20, 3.26)	0.52
3 vs 1+2 at AHI ≥15	1.43 (0.54, 3.82)	
**E-selectin (2+3 vs 1)**	**HR (95% CI)**	**p-value for interaction**
2+3 vs 1 at AHI <15	0.67 (0.19, 2.38)	**0.07**
2+3 vs 1 at AHI ≥15	3.31 (0.94, 11.72)	

Abbreviations: AHI: Apnea-Hypopnea Index; CI: Confidence Intervals; ICAM-1: Intercellular adhesion molecule-1; VCAM-1: Vascular cell adhesion molecule-1 HR: Hazard ratio; OSA: Obstructive Sleep Apnea

Adjusted for age, sex, smoking status, BMI, heart disease, ESS > 11, diabetes, CPAP adherence, and statins.

In contrast, E-selectin appeared to be more predictive in patients with OSA than patients without OSA. Specifically, when only OSA patients were considered, patients in the two higher tertiles had a rate of 1^st^ cardiovascular event of 10.9% compared to 5.4% in the 1^st^ tertile (adjusted HR = 2.21, CI: 0.87–5.66). Rates of events in patients without OSA was similar (or less) in the top two vs. lowest tertile (4.8 vs. 7.7%; HR = 0.34, 95% CI 0.04–2.62; p-value for interaction = 0.1). In patients with moderate to severe OSA, E-selectin was highly predictive of cardiovascular events, with rates of 14.2% and 4.9% in higher vs. lowest tertiles respectively (HR = 3.31, 95% CI: 0.94–11.72) with a significant interaction effect (p = 0.07).

### CPAP adherence and cardiovascular events

We assessed rates of events in patients prescribed CPAP (N = 134, S5 Table in S1 File). This number might be an underestimate of patients prescribed CPAP as patients may have been prescribed by their family doctor, or this data may not have been available in the record. The rate of events was 14.81% and 12.26% in the CPAP non-adherent and CPAP adherent groups, respectively. In fully adjusted models, there was no difference in the hazard ratio of cardiovascular events (S6 Table in S1 File); similarly to recent randomized trials in secondary cardiovascular events [23].

## Discussion

In our retrospective cohort study, we found that patients in the top two tertiles of ICAM-1 (i.e. above 816 ng/ml) were significantly more likely to suffer a CV event during eight years of follow (HR = 3.65, 95% CI 1.40–9.53). This effect was similar in patients with OSA, and in moderate to severe OSA (OR = 3.1 and 2.74, respectively). E-selectin (>36.4 ng/ml) was significantly associated with cardiovascular events in patients with moderate to severe OSA (HR = 3.31), but not in patients without OSA. VCAM-1 was not associated with events. This is the first study investigating the prognostic utility of adhesion molecules in a large suspected well-defined OSA population.

OSA and the consequent intermittent hypoxia lead to oxidative stress and activation of pro-inflammatory transcription factors, such as HIF-1α and NFκB, which are responsible for expression of ICAM-1, VCAM-1 and selectins [14]. This concept is supported by data from human and animal studies in OSA [24–28] including in intervention trials using CPAP [29] showing elevated levels of CAM in OSA patients and reductions with CPAP. Activation of CAM represents a potential pathway by which OSA leads to premature CVD. In addition, variability of CAM levels in OSA patients due to genetic and other factors might explain some of the variability in CV risk associated with OSA. For example, we have recently shown that E-selectin levels are affected not only by OSA severity, but also body mass index and particular genetic polymorphisms (e.g., single nucleotide polymorphism rs579459 of the ABO gene) [18].

Our results with respect to ICAM-1 are consistent with studies in other populations (i.e., that did not assess degree of OSA). In a case control study, Hwang et al compared 204 patients with incident CVD to 316 control subjects and found that patients in the highest quartile of ICAM-1 had a 5.53 higher odds of developing CVD (95% CI, 2.51–12.21) [16]. Similarly, Luc et al compared 317 men to 613 matched controls; adjusted baseline levels of ICAM-1 were associated with an increased relative risk(RR) for myocardial infarction (RR 1.34; 95% CI 1.07–1.67) over 5 years [30]. Our study extends these results to specific OSA populations with detailed PSG information and an 8-year retrospective follow-up. Our study shows that ICAM-1 levels in patients with suspected OSA sent to a sleep clinic are predictive of CV events even after controlling for confounders such as OSA severity (AHI). In addition, in the group of patients with low ICAM and without OSA, no cardiovascular events were seen suggesting these are a group who would be considered very low risk.

Our findings with respect to E-selectin are particularly intriguing. In the case control study by Hwang et al (referenced above), high levels of E-selectin (fourth quartile) were not significantly associated with incident coronary heart disease (OR = 1.6, 95% CI 0.78–3.3) [16]. Although it is likely that some of these patients in the study had OSA, in unselected populations E-selectin does not seem to be a robust marker of incident CV events. These results are consistent with our results in which we found that in the entire cohort, E-selectin was not a significant predictor of cardiovascular events. However, we found that E-selectin was predictive of events in patients with OSA and particularly predictive in patients with moderate to severe OSA, suggesting it is a biomarker particularly relevant and specific to these populations. This concept is consistent with our previous study in which E-selectin was more strongly associated with OSA severity than other adhesion molecules [13].

There are arguments that that might favor an increased role of E-selectin in OSA over the other adhesion molecules. First, local recruitment and rolling of leukocytes along the endothelial walls are primarily mediated by selectins, while the further migration and firm attachment of white blood cells are believed to be mediated by ICAM and VCAM once the endothelium is activated. Though speculative, this initial recruitment might be more important in OSA. In a previous study, we have also shown that E-selectin is associated with OSA more strongly than other CAM [31]. Specifically, in that study (which used almost the same individuals as in the current study), we showed that OSA severity was more strongly associated with E-selectin levels than other adhesion molecules. Furthermore, genetic determinants of E-selectin interact with OSA severity, potentially modulating cardiovascular risk [18].

In contrast, VCAM-1 was not significantly associated with cardiovascular events in the whole cohort (OR = 1.44, 95% CI 0.68–3.04, p = 0.34) nor in patients with OSA (OR = 1.23, 95% CI 0.55–2.73, p-value for interaction = 0.28). This is in agreement with the study by Hwang et al., where VCAM-1 was not associated with fatal and non-fatal CVD events (OR = 1.01, 95% CI 0.79–1.30) [16] in an unselected cohort. It could be speculated that the different role of VCAM-1 in the establishment of cardiovascular lesions, differences in surface receptors [32] and the limited number of events seen in our study could explain our null findings.

Our study has many strengths. These included the retrospective cohort design, use of objective outcomes for cardiovascular events (as opposed to self-reports), collection of inflammatory markers at the same time each day (morning fasting), use of PSG to assess OSA severity, and ability to adjust for a variety of confounders. In addition, this is the first study of CAM and CVD prognosis done in a suspected OSA population, which is of substantial clinical relevance.

However, we acknowledge that our study also has a number of limitations. First, we had a relatively small number of first events. Because we used a broad composite outcome, the severity of each individual event or procedure may vary. Also, this was a single center study, which limits the generalizability of these findings. Larger studies are needed to verify these findings and provide more robust information for calculation of sensitivity and specificity of these molecules in risk prediction. Larger cohorts would allow for the incorporation of other variables into predictive models (e.g. symptom clusters [4], comorbidities, advanced physiologic data [33], other biomarkers [8] such as CRP) using machine learning techniques. Moreover, we measured baseline levels of ICAM-1, VCAM-1 and E-selectin. This measurement at one point in time does not address possible fluctuations of adhesion molecules over time. Also, there is a risk for residual confounding from unmeasured confounders that weren’t included in our data collection and analysis. Finally, the CPAP adherence data was largely based on chart-review as opposed to objective CPAP downloads.

## Conclusions

Suspected OSA patients with elevated ICAM-1 levels are significantly more likely to experience a cardiovascular event in 8 years of follow up. This association remained significant after adjustment for clinically significant confounding factors (age, sex, smoking status, AHI, BMI, previous heart disease, CPAP use, diabetes and statins). There was a potential interaction between OSA severity and levels of E-selectin, in that E-selectin tended to be more predictive of CV events in patients with moderate/severe OSA. Although there is need for larger validation studies, ICAM-1 and E-selectin could be potentially useful to identify which OSA patients might be at increased risk of future CV events.

## Supporting information

S1 File (DOCX)
