# Supplementary material for: Circulating levels of cell adhesion molecules and risk of cardiovascular events in obstructive sleep apnea
Source: PLoS One. 2021 Jul 30;16(7):e0255306. doi: 10.1371/journal.pone.0255306 (PMC8323915; doi:10.1371/journal.pone.0255306)
Supplement: S1 File — (DOCX) [file pone.0255306.s001.docx]

**Supporting information**

The databases used through Population Data BC were Discharge Abstracts (DAD), Cardiac Services of BC (CSBC), Vital Statistics and the Consolidation file. DAD captures administrative, clinical and demographical data from the Ministry of Health on hospital discharges (including deaths, sign-outs and transfers). The ICD-10-CA codes were extracted from DAD. CSBC was used to provide information on PCI and CABG. Vital Statistics collects data on British Columbians birth, adoption, deaths, marriages and divorces. We collected year, month, day of death with underlying cause of death based on ICD-10-CA codes. The Consolidation File includes health insurance information (Ministry of Health Registration and Premium Billing file, Medical Services Plans and postal codes) and it was used to create censoring variables.

All inferences, opinions and conclusions drawn in this paper are those of the authors, and do not reflect the opinions or policies of the Data Stewards(s).

Data extracts were provided by the British Columbia Ministry of Health. These extracts are cited below in compliance with Population Data BC protocols.

Canadian Institute for Health Information [creator] (2018): Discharge Abstract Database (Hospital Separations). Population Data BC [publisher]. Data Extract. MOH (2018). <http://www.popdatabc.ca/data>

BC Vital Statistics Agency [creator] (2018): Vital Statistics Deaths. Population Data BC [publisher]. Data Extract BC Vital Statistics Agency (2018). <http://www.popdatabc.ca/data>

Cardiac Services BC Consolidation Registry [creator] (2018): Cardiac Services BC (CSBC). Population Data BC [publisher]. Data Extract Cardiac Services BC (2018). <http://www.popdatabc.ca/data>

**S1-Table. Cardiovascular Causes of Death Codes**

| **ICD10 Code** | **Cause of Death** |
| --- | --- |
| E785 | Hyperlipidemia |
| I219 | Myocardial Infarction |
| I251 | Atherosclerotic Heart Disease |
| I259 | Chronic Ischemic Heart Disease, Unspecified |
| I514 | Myocarditis, Unspecified |
| *Abbreviations: ICD: International Classification of Diseases* | |

**S2-Table. ICD10/CCI Codes for Endpoints**

| **Outcome** | **ICD10** | **Diagnosis Type** |
| --- | --- | --- |
| AFIB | I48 | M (most responsible for the patient’s stay in a facility) |
| CHF | I50 | M (most responsible for the patient’s stay in a facility) |
| MI | I21., I22 | Any |
| Stroke | I60-I64 | Any |
| Unstable Angina | I200, I249 | Any |
| Ventricular Tachycardia | I472 | Any |
| **Procedures** | **CCI** |  |
| CABG | 1IJ76 |  |
| PCI | 1IJ50, 1IJ57GQ |  |
| Cardioversion | 1HZ09 |  |
| Defibrillator | 1HZ53GRFS, 1HZ53HAFS, 1HZ53LAFS, 1HZ53SYFS |  |
| Pacemaker | 1HZ53GRNM, 1HZ53LANM, 1HZ53QANM, 1HZ53GRNK, 1HZ53LANK, 1HZ53QANK, 1HZ53GRNL, 1HZ53LANL, 1HZ53QANL |  |
| Cardiac Resynchronization Therapy Pacemaker | 1HZ53GRFS, 1HZ53HAFS, 1HZ53LAFS, 1HZ53SYFS |  |
| Definitions: The events included stroke, myocardial infarction, primary heart failure, acute coronary syndrome, ventricular tachycardia, atrial fibrillation, percutaneous coronary intervention (PCI) and coronary artery bypass graft (CABG). Stroke was defined as nontraumatic subarachnoid hemorrhage (I60), nontraumatic intracerebral hemorrhage (I61), cerebral infarction (I63) stroke not specified as hemorrhage or infarction(I64). Transient ischemic attacks (TIA) were not included in the stroke diagnosis. Myocardial infarction was defined as acute myocardial infarction including all transmural sites (I21), and subsequent myocardial infarction (I22). Heart Failure was defined as primary acute decompensated heart failure (I50). Ventricular tachycardia was defined using code ICD-10-CA I47.2. Atrial fibrillation was defined as paroxysmal atrial fibrillation (I48.0). Acute coronary syndrome included unstable angina (I200), unspecified acute ischemic heart disease (I248, I249), angina pectoris with documented spasm (i201), other angina (I208), unspecified angina pectoris (I209), coronary thrombosis not resulting in myocardial infarction (I240), and Dressler’s syndrome (I241). We only included deaths with underlying cardiovascular causes. Death causes included hyperlipidemia (E 785), myocardial infarction (I219), atherosclerotic heart disease (I251), chronic ischemic heart disease (I259), and myocarditis (I514). | | |

**S3-Table – Hazard Ratios dividing models by quartiles and tertiles**

| **Unadjusted Quartiles** | **HR (95% CI)** | **p-value** | **Unadjusted Tertiles** | **HR (95% CI)** | **p-value** |
| --- | --- | --- | --- | --- | --- |
| **2 vs 1** | 5.72 (1.27, 25.81) | 0.02 | **2 vs 1** | 3.27 (1.20, 8.91) | 0.02 |
| **3 vs 1** | 6.41 (1.43, 28.64) | 0.01 | **3 vs 1** | 3.25 (1.18, 8.93) | 0.02 |
| **4 vs 1** | 5.38 (1.18, 24.54) | 0.03 | **-** |  |  |
| **Tertile 2+3+4 vs 1** | **HR (95% CI)** | **p-value** | **Tertile 2+3 vs 1** | **HR (95% CI)** | **p-value** |
| **Unadjusted** | 5.84 (1.40, 24.32) | 0.01 | **Unadjusted** | 3.26 (1.27, 8.37) | 0.01 |
| **Fully Adjusted*** | 6.51 (1.53, 27.70) | 0.01 | **Fully Adjusted*** | 3.65 (1.40, 9.53) | 0.008 |
| *Adjusted for Age, Sex, Smoking Status, AHI, BMI, Heart Disease, ESS > 11, Diabetes, CPAP Adherence, Statin Use | | | | | |

**S4-Table – Univariate Associations with Cardiovascular Events**

| **Characteristic** | **OR (95% CI)** | **p-value** |
| --- | --- | --- |
| Age | 1.10 (1.06, 1.14) | <0.0001 |
| BMI | 1.00 (0.96, 1.06) | 0.79 |
| Sex**^+^** | 1.78 (0.79, 4.01) | 0.15 |
| AHI | 1.01 (0.99, 1.02) | 0.10 |
| Heart Disease* | 1.79 (0.86, 3.72) | 0.11 |
| Diabetes**^++^** | 1.99 (0.71, 5.51) | 0.18 |
| Smoking**^+++^** | 1.65 (0.60, 4.54) | 0.32 |
| Statin**^++++^** | 2.27 (1.08, 4.76) | 0.02 |
| ESS > 11**^+++++^** | 1.20 (0.59, 2.44) | 0.27 |
| ***Abbreviations***: AHI: Apnea-Hypopnea Index; BMI: Body mass index; CI: Confidence Intervals; ESS: Epworth Sleepiness Scale; OR: Odds ratio;; *******Heart disease included: Hypertension, Myocardial Infarction; Cardiac Arrhythmias, Angina, and Congestive Heart Failure; **^+^**Male versus Female; **^++^**With vs without; **^+++^**Current smokers versus not currently smoking; **^++++^**Users vs. not users; **^+++++^** Above 11 (sleepy) vs. below (non-sleepy). | | |

**S5-Table. Patient Characteristics by CPAP Adherence**

| *Baseline characteristics* | *Not Adherent (n=27)* | *Adherent (n=107)* | *p-value* |
| --- | --- | --- | --- |
| Age - mean ± SD | 54.5 ± 10.1 | 52.8 ± 10.1 | 0.44 |
| Sex (Male) – n (%) | 17 (63) | 81 (76.4) | 0.16 |
| Body Mass Index - mean ± SD | 30.77 ± 5.71 | 33.19 ± 6.99 | 0.10 |
| AHI (events/hr) – median (IQR) | 13.10 (7.90,25.60) | 27.80 (15.60,46.10) | **0.02** |
| AHI Category |  |  | **0.02** |
| Control - n (%) | < 6 | < 6 | . |
| Mild OSA - n (%) | 13 (48.1) | 21 (19.6) | . |
| Moderate OSA - n (%) | 7 (25.9) | 31 (29) | . |
| Severe OSA - n (%) | 6 (22.2) | 50 (46.7) | . |
| AHI Severity |  |  | **0.02** |
| Non-Severe - n (%) | 21 (77.8) | 57 (53.3) | . |
| Severe - n (%) | 6 (22.2) | 50 (46.7) | . |
| % Time below 90% SaO2 – median (IQR) | 0.14 (0.08,1.62) | 1.50 (0.19,7.40) | 0.08 |
| > 5 Minutes below 90% SaO2 - n (%) | 7 (25.9) | 51 (48.6) | **0.03** |
| ESS > 11 - n (%) | 11 (40.7) | 45 (42.1) | 0.90 |
| Heart Disease******* - n (%) | 7 (25.9) | 29 (27.1) | 0.90 |
| Smoker^+^ - n (%) | < 6 | 12 (11.2) | 0.24 |
| Diabetes - n (%) | < 6 | 10 (9.3) | 0.78 |
| Ethnicity Group |  |  | 0.84 |
| Caucasian - n (%) | 22 (81.5) | 86 (81.1) | . |
| Asian - n (%) | < 6 | 11 (10.4) | . |
| Other - n (%) | < 6 | 9 (8.5) | . |
| Statin User - n (%) | < 6 | 21 (20.6) | 0.81 |
| ***Abbreviations***: AHI: Apnea-Hypopnea Index; BMI: Body mass index; ESS: Epworth Sleepiness Scale; IQR: Interquartile range; SD: Standard deviation; SaO_2_: Oxygen Saturation; *******Heart disease included: Hypertension, Myocardial Infarction; Cardiac Arrhythmias, Angina, and Congestive Heart Failure. ^+^Current Smokers. | | | |

**S6-Table. Unadjusted and Adjusted Hazard Ratio for CPAP Adherence and Cardiovascular Events.**

|  | **HR (95% CI)** | **p-value** |
| --- | --- | --- |
| **Unadjusted** | 0.86 (0.28, 2.63) | 0.79 |
| **Fully Adjusted***** | 0.92 (0.26, 3.26) | 0.90 |

***Adjusted for Age, Sex, Smoking Status, AHI, BMI, Heart Disease, ESS > 11, Diabetes, CPAP adherence and Statin Usage

**S7-Table. *Patient Characteristics by OSA Severity (n=418)***

|  | ***No OSA (AHI <5)*** | ***Mild OSA (AHI ≥5-15)*** | ***Moderate OSA (AHI ≥15-30)*** | ***Severe OSA (AHI≥ 30)*** |  |
| --- | --- | --- | --- | --- | --- |
| *Baseline characteristics* | *(n=69)* | *(n=126)* | *(n=115)* | *(n=107)* | *p-value* |
| Age - mean ± SD | 45.2 ± 11.4 | 50.5 ± 11.8 | 51.8 ± 10.1 | 53.4 ± 11.4 | **<0.001** |
| Sex (Male) - n (%) | 39 (56.5) | 80 (63.5) | 80 (69.6) | 87 (82.1) | **0.002** |
| Body Mass Index (kg/m^2^) - mean ± SD | 30.748 ± 7.175 | 30.670 ± 6.485 | 32.193 ± 6.260 | 33.140 ± 6.219 | **0.01** |
| Body Mass Index Category |  |  |  |  | **0.010** |
| Normal - n (%) | 14 (20.3) | 21 (16.7) | 12 (10.4) | 6 (5.6) | . |
| Overweight - n (%) | 25 (36.2) | 49 (38.9) | 36 (31.3) | 25 (23.4) | . |
| Class 1 Obesity - n (%) | 15 (21.7) | 30 (23.8) | 32 (27.8) | 45 (42.1) | . |
| Class 2 Obesity - n (%) | 8 (11.6) | 13 (10.3) | 18 (15.7) | 18 (16.8) | . |
| Class 3 Obesity - n (%) | 7 (10.1) | 13 (10.3) | 17 (14.8) | 13 (12.1) | . |
| % Time below 90% SaO2 - _-_ median (IQR) | 0.08 (0.00,0.22) | 0.17 (0.04,0.66) | 0.71 (0.09,2.30) | 3.82 (0.69,12.64) | **<0.001** |
| ESS > 11 - n (%) | 23 (33.3) | 35 (27.8) | 36 (31.3) | 31 (29) | 0.87 |
| Heart Disease* - n (%) | 11 (15.9) | 32 (25.4) | 22 (19.1) | 25 (23.4) | 0.51 |
| Smoker^+^ - n (%) | 7 (10.1) | 7 (5.6) | 11 (9.6) | 12 (11.2) | 0.60 |
| Diabetes n (%) | < 6 | 9 (7.1) | < 6 | 13 (12.1) | 0.29 |
| Ethnicity Group |  |  |  |  | 0.58 |
| Caucasian - n (%) | 53 (79.1) | 101 (82.1) | 93 (81.6) | 80 (76.2) | . |
| Asian - n (%) | < 6 | 9 (7.3) | 9 (7.9) | 14 (13.3) | . |
| Other - n (%) | 10 (14.9) | 13 (10.6) | 12 (10.5) | 11 (10.5) | . |
| Statin User - - n (%) | 7 (10.1) | 25 (20.2) | 22 (19.5) | 26 (24.8) | 0.12 |
| Adhesion Molecules |  |  |  |  | . |
| E Selectin (ng/ml) | 42.22 (31.70,57.27) | 39.98 (30.18,53.49) | 42.93 (33.74,57.82) | 49.78 (38.03,64.92) | **0.003** |
| VCAM -1 (ng/ml) | 868.12 (761.27,1048.05) | 921.03 (779.48,1041.74) | 907.28 (775.17,1062.04) | 886.07 (784.36,1031.66) | 0.45 |
| ICAM -1 (ng/ml) | 73.04 (60.43,94.43) | 69.39 (50.01,89.42) | 72.96 (48.20,90.25) | 73.18 (53.49,97.30) | 0.27 |
| ***Abbreviations***: AHI: Apnea-Hypopnea Index; BMI: Body mass index; ESS: Epworth Sleepiness Scale; ICAM-1: Intercellular adhesion molecule-1; IQR : Interquartile range; SD: Standard deviation; SaO_2_: Oxygen Saturation; VCAM-1: Vascular Cell Adhesion Molecule-1; *******Heart disease included: Hypertension, Myocardial Infarction; Cardiac Arrhythmias, Angina, and Congestive Heart Failure. ^+^Current Smokers. | | | | | |
